# A Single Tri-Epitopic Antibody Virtually Recapitulates the Potency of a Combination of Three Monoclonal Antibodies in Neutralization of Botulinum Neurotoxin Serotype A

**DOI:** 10.3390/toxins10020084

**Published:** 2018-02-15

**Authors:** Jianlong Lou, Weihua Wen, Fraser Conrad, Qi Meng, Jianbo Dong, Zhengda Sun, Consuelo Garcia-Rodriguez, Shauna Farr-Jones, Luisa W. Cheng, Thomas D. Henderson, Jennifer L. Brown, Theresa J. Smith, Leonard A. Smith, Anthony Cormier, James D. Marks

**Affiliations:** 1 Department of Anesthesia and Perioperative Care, University of California, San Francisco Rm 3C-38, San Francisco General Hospital, 1001 Potrero Ave, San Francisco, CA 94110, USA; weihua.wen@ucsf.edu (W.W.); fraser.conrad@ucsf.edu (F.C.); fanqimeng@gmail.com (Q.M.); jianbodong@gmail.com (J.D.); zhengda.sun@ucsf.edu (Z.S.); MariaConsuelo.Garcia@ucsf.edu (C.G.-R.); Shauna.farr-jones@ucsf.edu (S.F.-J.); 2 Western Regional Research Center, Agricultural Research Service, United States Department of Agriculture, Albany, CA 94710, USA; luisa.cheng@ars.usda.gov (L.W.C.); Thomas.henderson@ars.usda.gov (T.D.H.); 3 Molecular and Translational Sciences Division, United States Army Medical Institute of Infectious Disease, Fort Detrick, MD 21702, USA; jennifer.l.brown436.ctr@mail.mil (J.L.B.); terrys2much@comcast.net (T.J.S.); 4 U. S. Army Medical Research and Materiel Command, Fort Detrick, MD 21702, USA; leonard.a.smith@comcast.net; 5 Department of Pathology, University of California, San Francisco, ZSFG/UCSF, Bldg 3/ Rm 211, 1001 Potrero Ave., San Francisco, CA 94158, USA; Anthony.Cormier@ucsf.edu

**Keywords:** bi-epitopic, tri-epitopic, multivalent antibody, recombinant monoclonal antibody, botulinum neurotoxin, antitoxin, botulism, mouse neutralization assay, negative-stain electron microscopy, kinetic-exclusion analysis

## Abstract

The standard of treatment for botulism, equine antitoxin, is a foreign protein with associated safety issues and a short serum half-life which excludes its use as a prophylactic antitoxin and makes it a less-than-optimal therapeutic. Due to these limitations, a recombinant monoclonal antibody (mAb) product is preferable. It has been shown that combining three mAbs that bind non-overlapping epitopes leads to highly potent botulinum neurotoxin (BoNT) neutralization. Recently, a triple human antibody combination for BoNT/A has demonstrated potent toxin neutralization in mouse models with no serious adverse events when tested in a Phase I clinical trial. However, a triple antibody therapeutic poses unique development and manufacturing challenges. Thus, potentially to streamline development of BoNT antitoxins, we sought to achieve the potency of multiple mAb combinations in a single IgG-based molecule that has a long serum half-life. The design, production, and testing of a single tri-epitopic IgG1-based mAb (TeAb) containing the binding sites of each of the three parental BoNT/A mAbs yielded an antibody of nearly equal potency to the combination. The approach taken here could be applied to the design and creation of other multivalent antibodies that could be used for a variety of applications, including toxin elimination.

## 1. Introduction

Botulism, a disease caused by botulinum neurotoxin (BoNT) intoxication, is characterized by flaccid paralysis, which if not rapidly fatal requires prolonged hospitalization in an intensive-care unit and mechanical ventilation. The mainstay of clinically proven treatment for botulism is therapeutically administered antitoxin, made by the immunization of human volunteers or by hyperimmunizing horses. Equine antitoxin [1,2] and human immune globulin [3,4] are currently used to treat adult and infant botulism, respectively. Human botulism-immune globulin is produced by plasmaphersing immunized laboratory personnel who are at risk of exposure to BoNT, and its production is not scalable. The current generation of equine antitoxin (BAT) is a Fab’_2_ polyclonal antibody that is not renewable, has associated hypersensitivity reactions, including cardiac arrest and serum sickness, and has a short serum half-life resulting in the potential for the relapse of botulism after treatment [5,6]. An alternative to serum-derived antitoxins is recombinant monoclonal antibody (mAb)-based antitoxin. While single mAbs do not neutralize BoNT with the requisite potency, combinations of two mAbs increase potency over a single mAb, with more potent neutralization seen with a combination of three mAbs binding non-overlapping epitopes [7].

We have generated a series of combinations of three mAbs that bind all toxin subtypes tested of BoNT/A, /B, /C, /D, /E, /F or /HA and that result in highly potent BoNT neutralization in mice [7,8,9]. The BoNT/A three-mAb combination (NTM-1631, formerly XOMA 3AB) has been tested in a Phase 1 clinical trial with no serious adverse events reported [10] and clinical development of the antibody combinations for other serotypes is ongoing (NCT02779140, B and NCT03046550, C/D). This oligoclonal strategy results in a highly potent drug for therapeutic and prophylactic use. However, a challenge for antibody combination therapeutics is the need to individually manufacture each component, combine them into a compatible formulation, and then assess the quality and stability of each component in the mixture individually and in combination. For example, a drug to treat the three most common serotypes (A, B and E) of BoNT would consist of nine antibodies that would need to be individually produced, purified, co-formulated, and qualified. As more serotypes are covered in the final drug product, the complexity of the product and the necessary analytics increases. An alternative would be to develop a single antibody-based molecule capable of binding multiple BoNT epitopes that could recapitulate the potency of mAb combinations. 

Here, we show that such single bi-epitopic mAbs (BeAb) and tri-epitopic mAbs (TeAb) can be generated that have similar BoNT binding and neutralization as the combination of two or three BoNT/A mAbs. Design of the TeAb was informed by first designing and testing a bi-epitopic antibody (BeAb) based on dual-variable domain (DVD) mAbs [11]. The BeAb and TeAb were designed to contain a single human Fc to ensure a long serum half-life. Such multi-specific mAbs may offer an alternative to mAb combinations as antitoxins, taking advantage of the synergistic effect of several mAbs in vivo and simplifying production, analysis, and preclinical and clinical development by making a single molecular entity.

## 2. Results and Discussion

### 2.1. Design of Bi-Epitopic (BeAb) and Tri-Epitopic Antibodies (TeAb)

**Figure 1:**
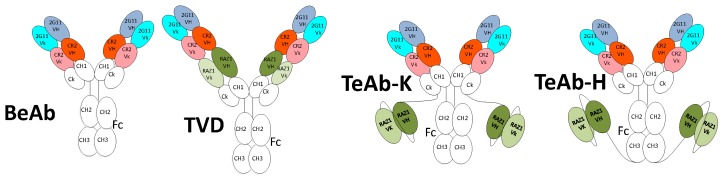
Cartoon of bi-epitopic antibody (BeAb) and three different V-domain arrangements of tri-epitopic antibody (TeAb) formats. The BeAb has the 2G11 Fv as the most external Fv while the CR2 Fv is internal. The three TeAbs are based on the BeAb Fv configuration with the addition of the RAZ1 Fv to the C-terminus of the CR2 V-domains in the BeAb (TVD) or as a single-chain Fv (scFv) fused to either the C-terminus of the kappa constant domain (TeAb-K) or the C-terminus of the Fc CH3 domain (TeAb-H).

The bi-epitopic antibody (BeAb, Figure 1A) was designed based on the DVD construct previously described [11] and contains the variable domains of mAbs 2G11 [12] and CR2 [13] connected to human IgG1/kappa constant domains (Figure 1). 2G11 binds an epitope on the BoNT/A light-chain domain-translocation domain (LC-H_N_) and CR2 bind an epitope on the N-terminal subdomain (H_CN_) of the BoNT/A binding domain (H_C_) [14]. The tri-epitopic antibody constructs (TeAbs) were designed based on the BeAb format, as shown in Figure 1. For these constructs, the RAZ1 mAb [13] variable regions were either added as a variable fragment (Fv) to the N-terminus of the BeAb or as single chain Fv fused to either the C-terminus of the kappa constant domain (TeAb-K) or to the C-terminus of the Fc CH3 domain (TeAb-H). RAZ1 binds an epitope on the C-terminal subdomain (H_CC_) of the BoNT/A binding domain (H_C_) [14].

### 2.2. Initial Characterization of the BeAb

**Figure 2:**
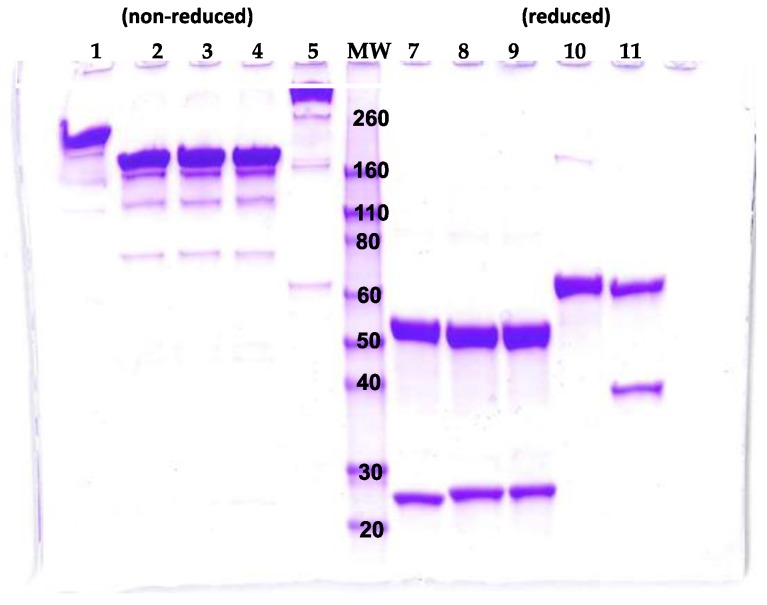
Sodium dodecyl sulfate polyacrylamide gel electrophoresis (SDS-PAGE) analysis of BeAb, and TeAb-K. Purified BeAb, IgG, and TeAb-K were run under non-reducing (lane 1–5; Lane 1: BeAb; Lane 2: RAZ1 mAb; Lane 3: CR2 mAb; Lane 4: 2G11 mAb; Lane 5: TeAb-k) or reducing (lane 7–11; Lane 7: RAZ1 mAb; Lane 8: CR2 mAb; Lane 9: 2G11 mAb; Lane 10: TeAb-k; Lane 11: BeAb) conditions. MW makers (lane 6) are at 260, 160, 110, 80, 60, 50, 40, 30, 20 kDa.

The BeAb was expressed from stably transfected CHO cells and purified by Protein G affinity chromatography with yields of approximately 10 mg/L of culture supernatant. Under non-reducing conditions, the BeAb ran at the expected size of approximately 200 kDa, larger than the predicted size of the 150 kDa 2G11 and CR2 IgG, and smaller than the TeAb (Figure 2). Under reducing conditions, the BeAb light chain with the additional V-domain (Vk) and heavy chain with the additional V-domain (V_H)_ ran at the expected sizes (37.5 kDa and 62.5 kDa, respectively). 

#### 2.2.1. Affinity of the BeAb for Botulinum Neurotoxin (BoNT/A)

Solution monovalent equilibrium dissociation constants (K_D_) of 2G11 and CR2 binding sites of the BeAb for antigen were measured using flow fluorimetry in a KinExA (Sapidyne Instruments Inc., Boise, ID, USA) and specific recombinant LC-H_N_ and H_CN_ domains, respectively (Figure S1A). The use of the domains allows accurate measurement of the monovalent K_D_ of one of the binding sites without the influence of binding of the second binding site. The BeAb bound the 2G11 epitope with an affinity comparable to the affinity of the parental 2G11 IgG (K_D_ 2G11 IgG = 1.1 × 10^−11^ M and K_D_ BeAb 2G11 binding site = 2.11 × 10^−11^ M, Table 1). In contrast, the BeAb bound the CR2 binding site with a K_D_ of 1.0 × 10^−8^ M, 38-fold higher (lower affinity) than the CR2 IgG. The lower affinity was largely the result of a 10-fold slower association rate (*k_on_*), likely due to steric hindrance from the 2G11 binding site (Figure 1) [15]. The affinities of the BeAb CR2 and 2G11 binding sites were also measured for the BoNT/A1 holotoxin using flow fluorimetry in a KinExA. In the case of holotoxin, different dimer, trimer or tetramer BeAb states can exist, for example binding of a single BeAb to holotoxin at a single binding site (affinity), binding at both binding sites (avidity) and crosslinking of holotoxin (avidity) (Figure S1C). Use of single mAb CR2 or 2G11 capture for the KinExA measurement, however, ensures that the measurement reflects the impact of these different binding modes specifically on the CR2 or 2G11 binding sites.

Using holotoxin, the BeAb bound the 2G11 binding site with a K_D_ approximately the same as the 2G11 IgG (K_D_ 2G11 IgG = 1.14 × 10^−11^ M and K_D_ BeAb 2G11 binding site = 1.28 × 10^−11^ M), as shown in Table 1. The approximately 2-fold lower K_D_ of the BeAb for holotoxin compared to CR2 IgG (higher affinity) than the CR2 IgG (Table 1) results from an almost 10-fold slower dissociation rate constant (*k_off_)*, likely reflecting avid binding from the 2G11 binding site. Differences in the binding of antibodies to the H_CN_ domain vs. holotoxin reflect the fact that the recombinant domain is not a perfect mimic of the CR2 epitope in the holotoxin [14]. These studies indicate that the BeAb recapitulates the high-affinity binding of CR2 and 2G11 in a single IgG-like molecule.

#### 2.2.2. In Vitro Functional Effects of the BeAb Compared to 2G11 and CR2 IgG

**Figure 3:**
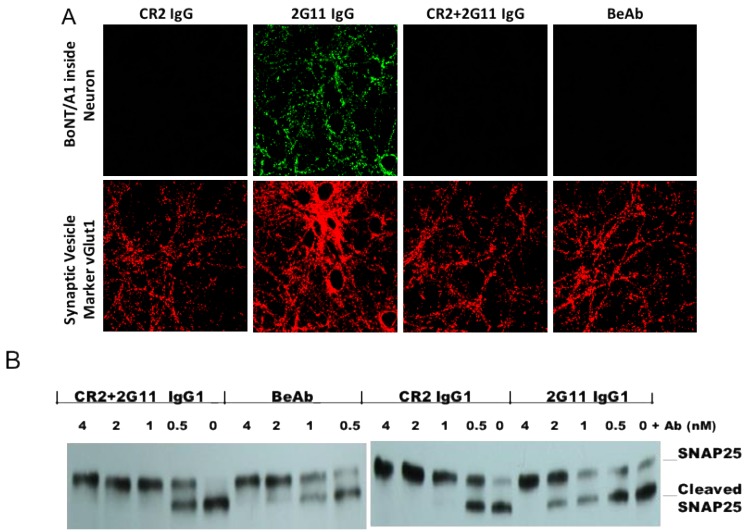
Blockade of BoNT/A uptake and SNAP-25 cleavage by BeAb, CR2 IgG and 2G11 IgG. (**A**) Ability of the indicated antibody to block uptake of BoNT/A into rat hippocampal primary neurons. Mixtures of BoNT/A1 and the indicated antibody shown were mixed, incubated with neurons for 30 min, stripped from the cell surface, the cells permeabilized and intra-neuronal BoNT/A detected using mAb(s), indicated. **B** Ability of the indicated antibody to block SNAP-25 cleavage. BoNT/A1 (1 nM) and the indicated antibody shown were mixed, incubated with neurons for 30 min, the cells lysed, and the lysate run on SDS-PAGE followed by Western blotting using an antibody specific for SNAP-25.

To ensure that the BeAb recapitulated the functional activity of CR2 and 2G11 in vitro, the ability of the BeAb, CR2 IgG, 2G11 IgG and the combination of CR2 + 2G11 IgG to prevent uptake of BoNT/A into neurons and cleavage of SNAP25 was compared (Figure 3). CR2 IgG blocked neuronal uptake of BoNT/A and, as a result, SNAP-25 cleavage, while 2G11 IgG did not block uptake and was not as potent as CR2 in inhibiting SNAP-25 cleavage. The BeAb recapitulated this effect of CR2, blocking BoNT/A uptake and more potently inhibiting SNAP-25 cleavage than 2G11 alone. CR2 binds to an H_C_ epitope that blocks the ability of BoNT/A to bind to its receptor [16].

### 2.3. Initial Characterization of the TeAb

The TVD, TeAb-K and TeAb-H were expressed from stably transfected CHO cells and purified by Protein G affinity chromatography with yields of approximately 5–10 mg/L of culture supernatant. The TVD and TeAb-H could not be purified to greater than 80% purity and the TVD also had a tendency to aggregate. These constructs were not studied further. Under non-reducing conditions, the TeAb-K ran close to its predicted size of 250 kDa (the 200 kDa of the BeAb component plus two 25 kDa scFv) (Figure 2). Under reducing conditions, four bands of approximately 62.5 kDa would be expected, two representing the 2G11 V_H_, the CR2 V_H_ and the CH1, CH2 and CH3 domains, and two representing the 2G11 Vk, the CR2 Vk, the Ck and the V_H_ and Vk of RAZ1.

#### Affinity of the TeAb for BoNT/A

Solution monovalent equilibrium dissociation constants (K_D_) of the individual RAZ1, 2G11, and CR2 binding sites of the TeAb-K for antigen were measured using flow fluorimetry in a KinExA and specific recombinant H_CC_, LC-H_N_, and H_CN_ domains, respectively (Figure S1B). The K_D_ values of the TeAb-K for each of the three epitopes on the holotoxin were also measured by incubating holotoxin with TeAb-K and capturing and measuring free holotoxin not bound to the epitope of focus using KinExA beads coated with either the RAZ1, 2G11, or CR2 IgG (Figure S1D). As described for the BeAb above, in the case of holotoxin, different dimer, trimer or tetramer TeAb states can exist, for example binding of a single TeAb to holotoxin at a single binding site (affinity), binding at two or more binding sites (avidity) and crosslinking of holotoxin (avidity) (Figure S1D). Use of single mAb CR2, 2G11 or RAZ1 capture for the KinExA measurement, however, ensures that the measurement reflects the impact of these different binding modes specifically on the CR2, 2G11 or RAZ1 binding sites.

Using recombinant BoNT domains, the TeAb-K bound the 2G11 and CR2 epitopes with K_D_ comparable to the K_D_ measured for these epitopes in the BeAb (Table 1). The TeAb-K bound the RAZ1 epitope with high affinity but with a K_D_ approximately 20-fold higher (lower affinity) than the RAZ1 K_D_ (K_D_ RAZ1 IgG = 1.33 × 10^−12^ M and K_D_ TeAb-K RAZ1 binding site = 2.55 × 10^−11^ M (Table 1). This may result from the conversion of the RAZ1 from a Fab in the IgG to a scFv in the TeAb-K [17]. The TeAb-K bound BoNT/A holotoxin with very high affinity at each of the three binding sites (RAZ1, CR2 and 2G11) with K_D_ ranging from 1.51 × 10^−11^ M to 2.57 × 10^−12^ M. These studies indicate that the TeAb-K recapitulates the high-affinity binding of the RAZ1, CR2 and 2G11 binding sites in a single IgG-like molecule.

### 2.4. BeAb and TeAb-K Neutralization of BoNT/A In Vivo

**Figure 4:**
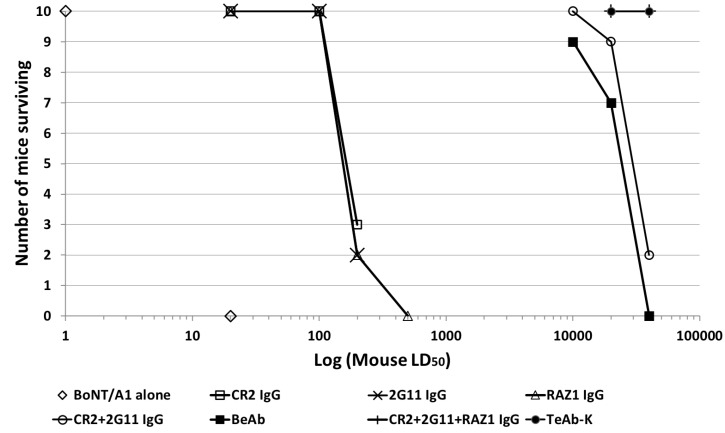

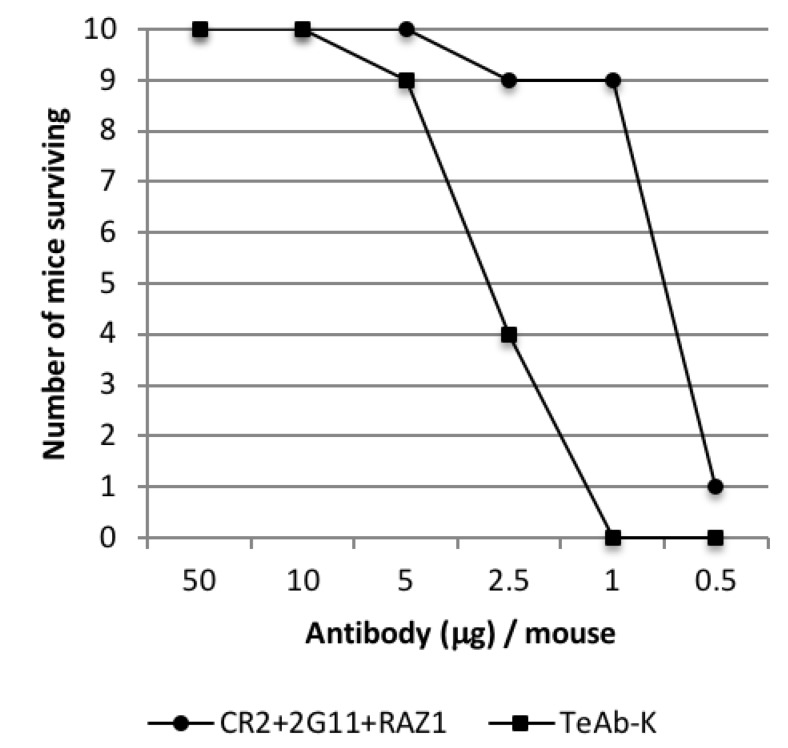
Antibody potency as assessed by the mouse neutralization assay (MNA). **Panel above**: relative potency of the BeAb and TeAb compared to CR2, 2G11 and RAZ1 IgG and their combinations. 50 μg of CR2, 2G11 or RAZ1 mAb, 25 μg each of the combination of CR2 + 2G11, 16.6 μg each of the combination of CR2 + 2G11 + RAZ1, 50 μg of BeAb or 50 μg of TeAb were mixed with the indicated amount of BoNT/A1 complex and injected intraperitoneally into cohorts of 10 mice, and the number of mice surviving at 5 days was determined. **Panel below**: relative potency of the TeAb compared to the combination of 2G11 + CR2 + RAZ1 IgG. 40,000 LD_50_ of BoNT/A1 was combined with either the TeAb or an equimolar combination of 2G11 + CR2 + RAZ1 IgG at the amount indicated, injected intraperitoneally into cohorts of 10 mice and the number of surviving mice was determined at 5 days.

The potency of the BeAb and TeAb in neutralizing BoNT/A in the mouse-neutralization assay (MNA) was compared to the potency of individual mAbs 2G11, CR2 and RAZ1, the combination of 2G11 and CR2 IgG and the combination of 2G11 + CR2 + RAZ1 IgG. Fifty μg of each of individual mAbs completely neutralized between 20 and 100 mouse LD_50_ in the MNA [18] (Figure 4). A combination of CR2 + 2G11 IgG (50 μg total antibody, 25 μg of each mAb) was approximately 100 times more potent than either single IgG, completely protecting mice challenged with 10,000 LD_50_ of BoNT/A (Figure 4). The BeAb (50 μg) was comparable in potency to the CR2 + 2G11 IgG combination, with 9/10 mice surviving challenge with 10,000 LD_50_ of BoNT/A compared to 10/10 mice surviving given the CR2 + 2G11 IgG combination. This result is consistent with previous studies of other BoNT/A mAbs showing mAb pairs neutralize BoNT/A much more potently than individual mAbs [7]. At a 40,000 LD_50_ challenge with BoNT/A, only 0/10 and 2/10 mice survived for mice receiving the BeAb or the 2G11 + CR2 IgG combination, respectively (Figure 4). In contrast, 50 μg of either the TeAb or the 50 μg total of the combination of 2G11 + CR2 + RAZ1 (16.6 μg of each mAb) IgG completely protected mice challenged with 40,000 BoNT/A LD_50_. To determine the potency of these antibodies, decreasing amounts of either the TeAb or the 2G11 + CR2 + RAZ1 IgG combination were mixed with 40,000 LD_50_ of BoNT/A and survival after five days was determined. The combination of 2G11 + CR2 + RAZ1 IgG or the TeAb were approximately 100-fold and 20-fold more potent than the 2G11 + CR2 IgG combination and approximately 200-fold and 40-fold more potent than the BeAb, respectively (Figure 4). The ED_50_ for the 2G11 + CR2 + RAZ1 IgG combination and TeAb vs. 40,000 BoNT/A LD50 were approximately 0.6 μg and 2.5 μg respectively, making the TeAb approximately four-fold less potent than the 2G11 + CR2 + RAZ1 IgG combination (Figure 4).

### 2.5. Mechanism of BeAb and TeAb BoNT/A Neutralization

**Figure 5:**
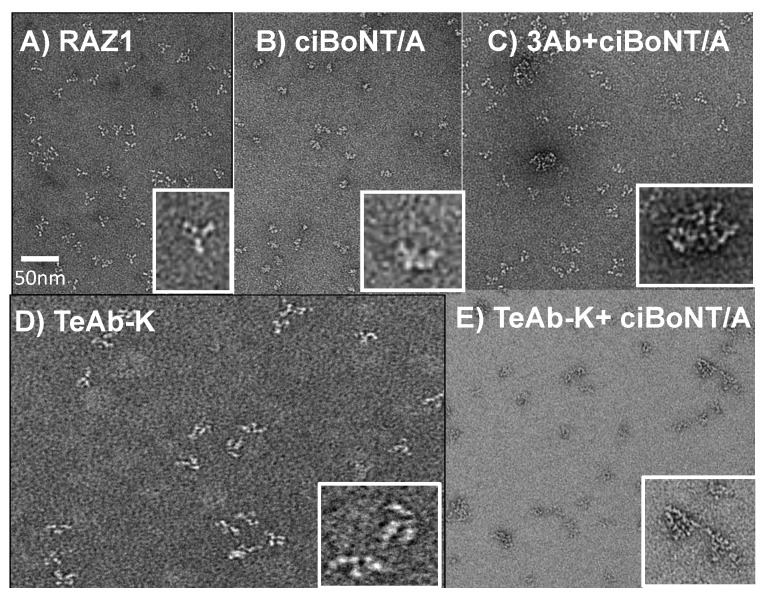
Negative-stain electron microscopy (NS-EM) images of TeAb-K immune complexes. Scale shown in (A) applies to all five panels, insert of each image is an enlargement of a single molecule or complex in greater detail. (**A**) RAZ1 IgG; (**B**) ciBoNT/A; (**C)** RAZ1 + 2G11 + CR2 + ci BoNT/A; (**D**) TeAb-K; (**E**) TeAb-K + ci BoNT/A.

One mechanism by which polyclonal antibody neutralizes BoNT is by accelerating its clearance through the liver by immune complex formation [19]. Negative-stain electron microscopy (NS-EM) was used to determine if the TeAb formed immune complexes with BoNT/A. For these experiments, catalytically inactive (ciBoNT/A, 17 nm average diameter) was used for safety reasons [20]. While the flexibility of the molecules and complexes precluded high-resolution images, the results provide insight into the types of complexes formed. In NS-EM, IgG alone exhibits the expected “Y” shape with an average diameter of 13 nm (Figure 5A). ciBoNT/A appears as a globular shape consistent with its known X-ray crystal structure [21] (Figure 5B). The three IgG CR2 + RAZ1 + 2G11 + ciBoNT/A produces complex structures; some unbound IgG are also visible (Figure 5C). The TeAb-K is “Y” shaped, but with additional domains, and is monodisperse with an average diameter of 20 nm (Figure 5D). The TeAb-K + ciBoNT/A forms more complex structures consistent with immune complexes containing what appears to be multiple TeAb-K and ciBoNT/A and with diameters between 26 to >100 nM Figure 5E).

**Figure 6:**
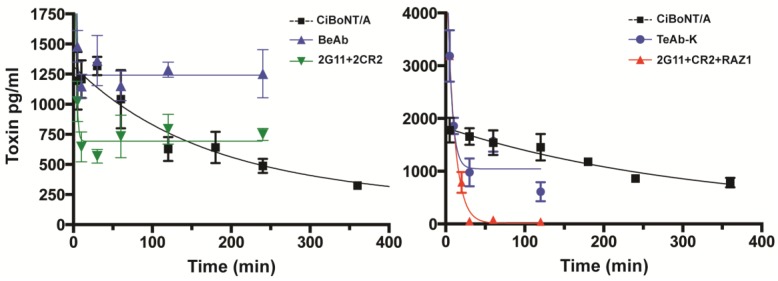
Clearance of ciBoNT/A in mice. **Left panel**: effect of BeAb or 2G11 + CR2 IgG on ciBoNT/A clearance. 5000 pg of of ciBoNT/A was injected iv into mice, followed 5 min later by iv injection of 10 μg of BeAb or an equimolar combination of mAbs CR2 and 2G11 IgG (5 μg of each). The ciBoNT/A concentration in mouse serum was measured at serial time points beginning 5 min after the BoNT/A injection using meso-scale discovery (MSD) based enzyme-linked immunosorbent assay (ELISA). CiBoNT/A without mAb was used as control. Black square: ciBoNT/A alone; blue triangles: BeAb; green triangles: 2G11 + 2CR2. **Right panel**: Effect of TeAb or 2G11 + CR2 + RAZ1 IgG on ciBoNT/A clearance. Mice were injected with 5000 pg of ciBoNT/A, an initial blood sample taken at 5 min and then mice injected with 10 μg of TeAb or the mAb combination CR2 + RAZ1 + 2G11, 3.3 μg of each). The BoNT/A concentration in mouse serum was measured at serial time points as described above. ciBoNT/A1 only without mAb was used as control. Square: ciBoNT/A alone; blue circles: TeAb-K; red triangles: 2G11, RAZ1, and CR2 combination. Each data point shown is the average value from three mice per data point.

The ability of the BeAb or TeAb to accelerate the clearance of ciBoNT/A from the circulation of mice was studied and compared to the effect of 2G11 + CR2 IgG and 2G11 + CR2 + RAZ1 IgG on ciBoNT/A clearance. These studies were performed by injecting 5000 pg of ciBoNT/A [20] into mice followed 5 min later by the injection of 10 μg of the BeAb, TeAb or IgG antibody combination (5 μg of each mAb for the double mAb combination and 3.3 μg of each mAb for the triple mAb combination) and measuring the serum concentration of ciBoNT/A at serial time points. Both the BeAb and the combination of CR2 + 2G11 IgG significantly slowed the clearance of ciBoNT/A from the circulation of mice compared to that of no antibody (Figure 6). We did not collect data at late enough time points to be able to calculate a BoNT-mAb half-life due to the reduced clearance. In contrast, both the TeAb-K and the combination of 2G11 + CR2 + RAZ1 IgG increased the clearance of ciBoNT/A from the circulation of mice compared to the control with no antibody, with the three mAb combination being more effective than the TeAb at increasing clearance. The two-phased clearance observed for the TeAb (an initial rapid phase followed by a slower terminal phase) would be consistent with a fraction of the immune complexes containing three Fc per BoNT (rapid clearance) and a fraction containing two or less per BoNT (slower clearance). Because of the multiple phases, we were unable to calculate a half-life for the TeAb-BoNT due to the multiple half-lives present, presumably due to the heterogeneity of the immune complexes. These clearance results are consistent with those observed by others. Antibody buffering and a reduction in clearance of antigen has been previously described for single mAbs binding an antigen and attributed to antigen bound to mAb picking up the long serum half-life of IgG [22]. Similarly, Mannik et al. [23] found that immune complexes containing one or two IgG persisted in the circulation, while those with more than two IgG were cleared rapidly. Montero-Julian et al. also found that IL-6 administered with one or two mAbs had its clearance reduced approximately 10 fold, and that clearance was returned to the rapid clearance observed for IL-6 only when three mAbs were administered [24].

## 3. Materials and Methods

### 3.1. Design, Cloning, Expression and Purification of BeAb and TeAb

The BeAb and three different TeAb expression-vector plasmids were constructed using the 2G11, CR2 or RAZ1- IgG1 N5KG1 expression vectors or their scFv leads in pYD2 vectors (Invitrogen, Thermo Fisher Scientific, Inc., Waltham, MA, USA) similar to those previously reported [25]. For BeAb construction, the second V-domain genes (Vk and V_H_ gene of 2G11) were sequentially inserted into the CR2-IgG expression vector, which was created using the IgG1 expression vector N5KG1 as previously reported [12], with sequence verification before transformation into mammalian CHO cells (ATCC, Manassas, VA, USA). The resulting construct has the 2G11 Vk and V_H_ domains fused to the C-terminus of the CR2 Vk and V_H_ domains respectively separated by a 14-amino acid linker1 of amino acid sequence TVAAPSVFIFPPSD for Vk, or linker2 ASTKGPSVFPLAPS for V_H_. Briefly, the Vk genes plus a linker were PCR- amplified from the mAb 2G11-IgG1 expression vector N5KG1 [12] using the following primer pairs:

BglIIinDraIIIoutVk5′primer: (5′-ATCACAGATCTCTCACCATGAGGGTCCCCGCTCAGCTCCTGGGGCTCCTGCTGCTCTGGCTCCCAGGTGCCCGATG-3′)
DraIIIinBsiWoutVklinker3′primer: (5′-ACTGCTCATCACATCGTGAGAAGATGAAGACAGATGGTGCAGCCACGGTACGTTTGATTTCC-3′)

The PCR-amplified Vk + linker1 fragment was double cut with *Bgl*II and *Dra*III restriction enzymes (New England Biolabs, Ipswich, MA, USA), purified and ligated into the mAb CR2-IgG1 expression vector backbone which was digested and purified similarly. The ligation product was used to transform *E. coli* DH5α competent cells, and vector plasmids containing the insertion were purified from the transformant for DNA sequencing. After sequence verification of the correct 2G11 Vk insertion into the mAb CR2-IgG1 plasmid from one of the transformants, the resulting vector was used for 2G11 V_H_ insertion to create the BeAb expression plasmid. The V_H_ genes with a second linker2 were polymerase chain reaction (PCR) amplified from the same mAb 2G11-IgG1 expression vector using a different pair of primers for the V_H_ + linker2 gene fragment:

SalIinMluIoutVH5′primer: (5′-GACCCGTCGACATGGGTTGGAGCCTCATCTTGCTCTTCCTTGTCGCTGTTGCTACCCGTGTCTTGTCCC-3′)
MluIinNheIout VH3′ tail primer: (5′-GGAGGAGGGTGCCAGACGCGTGACCGATGGGCCCTTGGTGCTTGCTGAGGAGACGG-3′)

The PCR-amplified V_H_ + linker2 gene fragment was double digested with *Sal*I and *Mlu*I restriction enzymes, purified, and subcloned into the plasmid from the previous step. The resulting plasmid was digested with same enzyme pairs before ligation and transformation. The ligation products were used to transform *E. coli* DH5α competent cells, and new plasmids were prepared from those transformants for sequencing. Both V-genes of mAb 2G11 and CR2 were confirmed to be in the BeAb expression vectors, and they were in frame with the leader sequence, and also in frame with the constant domain gene plus the designed linkers. This confirmation was achieved via sequencing the plasmid from upstream of the leader sequence and downstream of the constant domain genes using VHfor, VHback, Vkfor and Vkback primers as previously published [26] and additional primers as listed in Table S1. 

Three triepitopic antibodies (TeAbs) were created based on the BeAb construct with the 2G11 and CR2 V-domain at the N-terminus of the IgG. The third binding site (RAZ1 V-domains) was inserted as either the innermost tandem V-domains fused to the C-terminus of CR2 V-domains and the N-terminus of the Ck and CH1 domains (TVD) or as a scFv at the C terminus of the Ck (TeAb-K) or CH3 (TeAb-H) as illustrated in Figure 1. 

For the TVD gene construction, two additional linkers encoding 13 amino acids each were used to link the CR2 and RAZ1 domain V genes; The amino acid for the Vk linker3 is TVAAPSVFIFSRC and that for the V_H_ linker4 is ASTKGPSVTRVLS. Linker3 and linker4 genes were added to the RAZ1 V gene via PCR by using the IgG expression plasmid and primer pairs RAZBsiWIlinkerVk5′primer and Vk3′primer, or RAZNheIlinkerVH5′ primer and VH3′ primer (Table S1). The fragment with the additional linker was spliced to the BeAb V-genes before subcloning the entire three V-domain genes back into the IgG expression vector backbone. Briefly, the third V_H_ and Vk fragment for TVD creation was PCR amplified first from RAZ1 IgG expression plasmid to include all the following: “*Bsi*WI enzyme restriction site and linker3 and RAZ1 Vk + *CK1* genes with *Spe*I restriction site, or “*Nhe*I restriction site and linker4 and RAZ1 V_H_ gene plus partial *CH1* gene and *Age*I restriction site”, then digested one PCR product with *Bsi*WI and *Eco*RI to obtain a fragment containing the Vklinker3, and digested the other purified PCR product with *Nhe*I and *Age*I for a fragment containing the VHlinker4, and then ligated the fragments sequentially into the BeAb expression plasmid vector that was digested with same pair of enzymes to obtain the TVD expression plasmid, [12] followed by transformation of *E. coli* DH5α with ligation products, and sequence confirmation from individual transformants after plasmid preparation. All six V-genes of mAbs 2G11, CR2 and RAZ1 were confirmed to be in the TVD TeAb expression vector, and they were in frame with the leader sequence, and also in frame with the constant domain gene plus the designed linkers in the correct order. The TeAb expression plasmid was then used to transform CHO cells using similar procedures as published [26].

For TeAb-K expression-vector construction, the RAZ1 scFv gene was first PCR amplified from RAZ1-scFv pYD2 yeast expression vector [13], using primer pairs (Table S1) LinkerKspliceRAZ5′primer and TeAbKEcoRIin3′primer, which encodes partial (16 AA) of an extra linker5 and the stop codon immediately after the scFv gene plus the restriction sites *Eco*RI for subcloning. Linker5 is a 20-amino acid long sequence of SGGSTSGSGKPGSGEGSSGS and was designed to be located between the RAZ1 scFv domain and the C-terminus of the Ck domain. This fragment was than spliced with another fragment which was PCR amplified from the IgG expression vector using primer pairs (Table S1) of TeAbKBsiWIin5′primer and RAZ1linkerKsplice 3′primer containing the entire Ck domain gene and with *Bsi*WI restriction site and partial overlapping (11AA) linker5 sequence added for splicing. After splicing PCR amplification, the entire fragment was double digested with *Bsi*WI and *Eco*RI, then ligated into the BeAb expression plasmid backbone which had been digested with the same enzymes and purified. After ligation, the product was used to transform *E. coli* DH5α, and plasmid purified from individual transformants for sequence confirmation using primer TeAbKscfvSeqprimer. 

For TeAb-H expression, vector construction was also performed using a three-step PCR and splicing strategy. The RAZ1 scFv gene was first PCR amplified from RAZ1-scFv pYD2 yeast expression vector, using primer pairs (Table S1) LinkerHspliceRAZ5′primer and TeAbHBamHIin3′primer, which also encodes a partial (16 amino acids) linker5 and a stop codon immediately after the scFv gene, plus the restriction sites BamHI at the 3′ end for subcloning. This fragment was then spliced with another fragment which was PCR amplified from the same IgG expression vector using primer pairs (Table S1) of TeAbHSmaIin5′primer and RAZlinkerHsplice 3′primer containing the partial CH3 domain gene and with a *Sma*I restriction site at the 5′ end. The splicing product was used to transform *E. coli* DH5α, and plasmid prepared from individual transformants for sequence confirmation using sequencing primer ForTeAbscfvseq and primer4scfvconfirm.

All V-genes and each linker gene sequence in the BeAb or TeAb expression vectors were confirmed to be as designed, and in frame with the leader sequence and with the constant domain gene. Sequencing of the plasmid was performed from upstream of the leader sequence and downstream of the constant domain genes as described above. Stably-transfected CHO DG44 cells were selected using the G418 (Invitrogen, Thermo Fisher Scientific, Inc., Waltham, MA, USA) resistant marker as previously described [7]. The transfected cells were cultured and expanded into 1L spinner flask for BeAb or TeAb production. Supernatant from the cell cultures were collected and filtered with 0.22 µm filters before loading on to HiTrap Protein G HP columns (GE Healthcare, Little Chalfont, UK) for purification as previously described [12,13]. Purified BeAb or TeAb were quantified via sodium dodecyl sulfate polyacrylamide gel electrophoresis (SDS-PAGE) and A_280_ measurement and used immediately or stored at −80 °C prior to characterization. 

### 3.2. Binding Affinity Determinations Using KinExA Analysis

Equilibrium dissociation constants (K_D_) and kinetic association and dissociation rate constants (*k_on_*, *k_off_*) of BeAb and TeAb for holotoxin and for specific recombinant BoNT domains were measured using a KinExA 3200 flow fluorimeter (Sapidyne Instruments Inc., Boise, ID, USA) as previously described [13,27]. Binding affinity assays were performed in duplicate; equilibrium titration data were fit to a 1:1 reversible binding model using KinExA Pro Software (version 4.2.12, Sapidyne Instruments, Boise, ID, USA, 2016) to determine the K_D_. The exponential decrease in the concentration of free antigen as a function of time was fit to a standard bimolecular rate equation using the KinExA Pro software to determine the *k*_on_. The *k*_off_ was calculated from the product of *k_on_* × *K*_D_.

A schematic of the KinExA method is provided in Supplemental Figure S1. Solution interactions of BeAb and TeAb with specific BoNT domains are monovalent, and four binding states exist at equilibrium of which only the free domain can be captured by the mAb-coupled beads in the flow cell. Solution interactions of BeAb and TeAb with holotoxin are multivalent, and many more binding states with free epitopes can form and be captured by the beads, including complexes formed by avid binding and crosslinking. 

### 3.3. Inhibition of BoNT/A1 Cellular Uptake and SNAP25 Substrate Cleavage in Primary Neurons by IgG1 or BeAb

Rat hippocampal primary neurons were prepared as described [28]. BoNT/A1 cellular uptake inhibition by each individual mAb or BeAb or the combination of two mAbs were tested in 12- to 14-day-old primary neurons using BoNT/A1 toxin only without mAb as positive toxin entrance control. The treated neurons were harvested and divided into two parts, one for BoNT/A1 entrance visual inspection using confocal microscope, the other for SNAP25 digestion detection with SDS-PAGE-based endopeptidase assay, as published [29].

For microscopy, internalized BoNT/A1 toxin inside the neuron was detected using mAb 4E17.1 [26] and DyLight 488 conjugated goat anti-human IgG (H + L, Jackson ImmunoResearch, Catalog #109-485-003), then recorded with a confocal microscope (Nikon Eclipse C1si True Spectral Imaging, Nikon Instruments, Inc., Melville, NY, USA). Briefly, 10 nM BoNT/A1 (Metabiologics Inc., Madison, WI, USA) was mixed with 100 nM or less of individual mAb CR2 or 2G11 IgG1, an equimolar combination of CR2 and 2G11, or 100 nM or less BeAb in high potassium buffer (87 mM NaCl, 56 mM KCl, 1.5 mM KH_2_PO_4_, 8 mM NaHPO_4_, 1 mM CaCl_2_, and 0.5 mM MgCl_2_) for 30 min at room temperature. Each mixture was then added to individual wells of 12- to 14-day-old primary neurons incubated at 37 °C for 30 min before phosphate-buffered saline (PBS) washing and 10% goat serum blocking of non-specific binding. BoNT/A1 without antibody was used as a positive uptake and cleavage control. After fixing the treated neurons with freshly prepared 4% paraformaldehyde, permeabilization of fixed cells was done by incubation with 0.2% Triton X-100. The neurons were sequentially incubated with 1 μg/mL of mAb 4E17.1 for two hours and 5 μg/mL of goat anti-human IgG secondary Ab for one hour at room temperature. A polyclonal guinea pig antibody for vesicular glutamate transporter 1 (Millipore, AB5905), a synaptic vesicle marker, was also used to show the location of presynaptic terminals when necessary. After washing, the samples were mounted and sent for imaging analysis.

In the SDS-PAGE-based endopeptidase assay, the amount of intact and cleaved SNAP25 from the treated neurons was detected with antibody to SNAP25. Briefly, neurons treated with test antibody (CR2, 2G11 or BeAb) at the concentration of 0–4 nM mixed with 1 nM of BoNT/A1 in the Opti-MEM media were monitored. Cells were scraped off the dishes and protein was extracted by using radio immunoprecipitation assay (RIPA) lysis and extraction buffer with proteinase inhibitors (Thermo Scientific). After centrifugation at 13,400 × *g* for 20 min, the supernatant was then collected and protein concentration was determined by the absorbance at the 280 nm. The extracted protein from the cell lysate was loaded onto a 4–20% SDS-PAGE gradient gel for electrophoresis and the separated proteins were then transferred onto a polyvinylidene difluoride membrane (iBlot gel transfer device, Life Technologies). The membrane was blocked with 5% fat-free milk, then immunoblotted overnight with a primary antibody against SNAP-25 (Santa Cruz Biotechnology Inc., Dallas, TX, USA) at 4 °C, followed by incubation with horseradish peroxidase-conjugated anti-mouse secondary antibody (Jackson Immuno Research, West Grove, PA, USA) and visualization using SuperSignal West Femto kit (Thermo Scientific, Grand Island, NY, USA) and autoradiography using Kodak film (Kodak Co., Rochester, NY, USA). The scanned images were also analyzed by using NIH Image J software for semi-quantitation followed by Western blotting (Figure 3B).

### 3.4. Mouse Neutralization Assay of BoNT/A1 by IgG, BeAb or TeAb

The United States Army Medical Research Institute of Infectious Diseases (USAMRIID) Institutional Animal Care and Use Committee (IACUC) approved the animal care and use protocol to conduct the animal studies described in this sub-section and in Section 3.5, below. Research was conducted under an IACUC approved protocol in compliance with the Animal Welfare Act, Public Health Service (PHS) Policy, and other federal statutes and regulations relating to animals and experiments involving animals. The facilities where this research was conducted are accredited by the Association for Assessment and Accreditation of Laboratory Animal Care, International (AAALAC/I) and adheres to principles stated in the Guide for the Care and Use of Laboratory Animals, National Research Council, 2011. The specific national regulations and guidelines to which this animal care and use protocol adheres are the following: (1) 7 United States Code (USC), Sections 2131–2159, Chapter 54 “Animal Welfare Act”, and (Code of Federal Regulations (CFR)), Chapter 1, Subchapter A, Parts 1–4 “Animal Welfare Regulations”; (2) Health Research Extension Act of 1985, Public Law 99–158 “Animals in Research” and the PHS Policy in Humane Care and Use of Laboratory Animals; (3) Biosafety in Microbiological and Biomedical Laboratories, 5th Edition, National Institutes of Health (NIH), Human and Health Services Publication (Centers for Disease Control and Prevention (CDC)) 21–112. Studies performed at USAMRIID also complied with (1) Army Regulation 40–33 “The Care and Use of Animals in Department of Defense (DOD) Research, Development, Test and Evaluation or Training Programs” and (2) DOD Instruction 3216.01 “Use of Animals in DOD Programs”. DOD uses “The Guide for the Care and Use of Laboratory Animals”, 8th Edition, Institute for Laboratory Animal Research, National Research Council, as a guideline for evaluation and accreditation of program and it is based on the actual national regulations and guidelines for animal care and use programs. The animals used in this study were euthanized using carbon dioxide gas following the American Veterinary Medical Association (AVMA) Guidelines on Euthanasia.

All procedures involving animals performed at the United States Department of Agriculture (USDA) were reviewed and approved by the Institutional Animal Care and Use Committee of the USDA, Western Regional Research Center. Animal use protocol for BoNT mouse bioassays (Protocol #15-8) was approved by the Western Regional Research Center Institutional Animal Care and Use Committee (WRRC-IACUC) on 27 July 2015.

BeAb, TeAb, individual IgGs of 2G11, CR2 or RAZ1, or a combination of two or three IgGs (1–50 μg total antibody per mouse) were added to an indicated number of mouse LD_50_ of BoNT/A1 holotoxin (Metabiologics Inc. Madison, WI, USA) in gelatin phosphate buffer (pH 6.5) and incubated at room temperature for 30 min. For the mAb pairs, antibody was added in a 1:1 ratio; for triple mAb combinations, antibody was added in a 1:1:1 ratio. The mixtures were injected intraperitoneally (i.p.) into groups of 10 female (CD-1 mice weighing 16–22 g upon receipt at USAMRIID, or CFW, Swiss Webster at USDA Western Regional Research Center). Mice were observed at 4–8 h intervals after toxin challenge. Mouse survival was recorded for 5 days after injection.

### 3.5. Estimation of TeAb-K Complex Size and Composition Using Negative-Stain Electron Microscopy (NS-EM)

Purified antibody as individual IgG or BeAb or TeAb, ciBoNT/A, or antibody-toxin complexes were applied to glow-discharged 400 mesh carbon-coated copper grids, washed, stained with 0.75% (*w*/*v*) uranylformate, and aspirated to dryness as reported [30]. Images were taken on a Tecnai T12 electron microscope (FEI Company, Hillsboro, OR, USA) equipped with a LaB6 filament and operating at a 120-kV acceleration voltage. Micrographs were recorded using a 4K charge-coupled device camera (UltraScan 4000, Gatan Inc. Pleasanton, CA, USA), where one pixel equals 2.21 Å on the specimen.

### 3.6. Pharmacokinetic Studies of ciBoNT/A in the Presence of Antibodies

Groups of three Swiss Webster female mice (Charles River, Portage MI, weights 19–20 g) were injected intravenously (iv) via the lateral tail vein with 5000 pg ciBoNT/A and 10 µg of mAb (IgG1, BeAb or TeAb) per mouse at indicated times. MAbs were diluted in PBS (pH 7.2). Blood was collected from the submandibular site into serum collection tubes with gel separators (BD Biosciences). Samples were incubated on ice for at least 30 min before centrifugation at 3000× *g* for 10 min to separate sera from the cellular fraction. Sera were aliquoted and stored at −80 °C prior to analysis. 

The concentration of ciBoNT/A in sera was determined by an electrochemiluminescence (ECL) ELISA using a SECTOR Imager 2400 (MSD, Gaithersburg, MD, USA). ECL was performed as described previously with slight modification [31]. Briefly, 96 well plates were coated with anti-BoNT/A mAb 5A20.4 at 2 µg/mL and incubated overnight at 4 °C. Plates were blocked with tris-buffered saline (TBS) Tween with 3% milk (block buffer). Toxin standards were prepared in 50% normal mouse sera (MP Biomedicals, Santa Ana, CA, USA) and sera samples were diluted in PBS (1:1). Sample plates were incubated at 37 °C for 1 h. After washing, 30 µL of 2 µg/mL rabbit polyclonal anti-BoNT/A (Metabiologics, Madison, WI, USA) was added and the plates were incubated for 1 h at 37 °C. The plates were then washed and incubated for 1 h with 30 µL of a 1:1000 diluted Sulfo-Tag labeled anti-rabbit antibody conjugate, followed by Reading buffer (MSD). The biologic half-life of toxin or mAbs was determined by calculating two-phase exponential decay over time using the GraphPad Prism statistics software (Version 7.0, Graphpad Prism, Inc., La Jolla, CA, USA).

## 4. Discussion

We have partially reproduced within four-fold the increased BoNT neutralization potency of double or triple mAb combinations in a single IgG-based molecule by creating biepitopic (BeAb) and triepitopic (TeAb) antibodies. The BeAb was designed based on the previously described dual variable domain (DVD) IgG [11]. Using epitope specific BoNT/A domains, it was shown that the affinity of the outer variable domains maintained the same affinity for BoNT/A as the parental IgG 2G11, while the inner variable domains had an approximately 40-fold lower affinity compared to the parental CR2 IgG due primarily to a 12-fold slower *k_on_*. Such a decrease in affinity due to a reduction in *k_on_* for the inner-variable domains has been previously reported for a DVD binding TNFα [15] and could be partially overcome by linker redesign. Regardless, affinity of both the 2G11 and CR2 binding sites for BoNT/A was comparable to that of the parental IgG, despite the lower affinity for the CR2 binding site in the epitope specific domain. While the *k_on_* for the CR2 binding site for BoNT/A was 4.6-fold slower than the CR2 IgG, this was more than compensated by a 8.6-fold slower *k_off_* for the CR2 site in the BeAb compared to CR2 IgG. These findings suggest that the BeAb avidly bound to BoNT/A, either simultaneously binding a single BoNT/A at both the 2G11 and CR2 epitopes or cross-linking multiple BoNT/A molecules. This is likely due to the flexibility of the 2G11 and CR2 binding sites in the DVD format [32]. The BeAb blocked the uptake of BoNT/A and cleavage of SNAP25 in neuronal cells, and recapitulated the potency of the combination of the two parental IgGs in a mouse-protection assay, with neutralization ED_50_ between 20,000 and 40,000 LD_50_ with a 50 µg/mouse total antibody dose. The potency of the BeAb, like the mAb pair combination, was approximately 100 times greater than the individual mAbs.

Three TeAb constructs were designed based on the successful BeAb structure. Of these, only the TeAb-K, with the RAZ1 scFv fused to the C-terminus of the kappa light-chain constant domain was produced with sufficient purity and quantity for detailed characterization. The TeAb-K had comparable affinity for the 2G11 and CR2 epitopes as the BeAb as measured by the epitope specific domains. The affinity of the TeAb for the RAZ1 epitope was approximately 19-fold lower than RAZ1 IgG, likely due to conversion to the scFv format. Affinity for BoNT/A at the 2G11 and CR2 epitopes were 5.4 and 3.4 times higher than for the 2G11 and CR2 IgG and the reduction of affinity of the RAZ1 scFv was only 8.8-fold. These data, like for the BeAb, suggest avid binding of the TeAb to BoNT/A. The TeAb-K had an ED_50_ for a 40,000 LD_50_ dose of BoNT/A of approximately 2.5 μg, or about 40-fold more potent than the BeAb and 20-fold more potent than the combination of mAbs 2G11 and CR2. The TeAb, however, was approximately four-fold less potent in BoNT/A neutralization than the combination of 2G11 + CR2 + RAZ1 IgG.

mAb combinations can synergize by a number of different mechanisms to neutralize BoNT. First, binding of multiple mAbs can interfere with multiple steps in the intoxication pathway, such as receptor binding and cell entry, endosomal translocation or intracellular catalysis [33]. Second, binding of multiple mAbs can create an avid antigen leading to an increase in mAb functional affinity [7]. Third, binding of multiple mAbs leads to rapid Fc-mediated clearance via the liver [19]. Finally, the large size of immune complexes likely slows the trafficking of BoNT to its site of action. In the case of mAb pairs and the BeAb, BoNT clearance is in fact reduced compared to BoNT only, indicating that other mechanisms such as blockade of multiple BoNT receptor biding sites or slowing the trafficking of BoNT to its site of action. In contrast, the triple mAb combination and TeAb accelerate BoNT clearance, likely explaining why these molecules are more potent. Rapid antigen clearance requires the binding of at least three Fc to antigen [23,24]. This clearly occurs with the triple mAb combination leading to rapid clearance. In contrast, based on the pharmacokinetic curve, it appears that only a fraction of the TeAb binds in that manner, likely explaining the four-fold reduction in potency.

Multifunctional antibodies have entered preclinical as well as clinical development. Despite the significant progress on bi-specific antibody constructs [11,34,35,36,37,38], however, there are fewer reports of tri-specific antibodies [39,40,41,42,43,44]. The majority of the bi-specific constructs and all of the trispecific antibodies in these reports target at least two distinct proteins rather than three distinct epitopes on the same protein, as reported here. A recent report, however, describes a tri-specific antibody binding three distinct binding sites on the HIV-1 envelope. In this study, multi-specificity increases potency and allows broader clade neutralization and higher potency than individual mAbs [45]. Similarly, the tri-epitopic mAb reported here has greater potency than single mAbs and should provide broader subtype binding than any individual mAb. Such tri-epitopic mAb could have great utility in the rapid clearance and neutralization of a range of toxins besides BoNT, such as ricin, and other toxic molecules. Whether such bi-epitopic or tri-epitopic molecules prove to be reliable alternatives to antibody combinations remains to be proved. Potential challenges include immunogenicity due to linkers, decreased stability or propensity for aggregation, due to non-IgG like V-domain pairings and reduced serum half lives and potency compared to antibody combinations. 

## 5. Conclusions

In summary, we have described how the synergy observed by combining three BoNT/A mAbs can almost be reproduced in a single tri-epitopic IgG-like molecule. Potency is increased more than 2000-fold compared to single mAbs. While it is unproven as to how easily such triepitopic antibodies can be developed as therapeutics, they may provide an alternative to mAb combinations for the treatment and prevention of botulism.

## Figures and Tables

**Table 1:** Affinities and binding kinetics of the BeAb, TeAb-K and IgG for epitope specific botulinum neurotoxin (BoNT/A1) domains and BoNT/A1 holotoxin as measured by KinExA.

	BoNT/A1 Domain			BoNT/A1 Holotoxin		
mAb	K_D_ (pM)	*k_on_* (10^6^ M^−1^s^−1^)	*k_off_* (10^−6^ s^−1^)	K_D_ (pM)	*k_on_* (10^6^ M^−1^ s^−1^)	*k_off_* (10^−6^ s^−1^) ^c^
H_CN_ (CR2 epitope)						
CR2	263.49 (325.94–171.09) ^a^	4.681 (6.081–3.514) ^a^	1234	8.84 (13.39–5.31)	3.650 (2.988–4.417)	32.27
BeAb ^b^	10,040 (12,760–6790)	0.3986 (0.4957–0.3463)	4002	4.73 (6.31–3.44)	0.7907 (0.8688–0.7181)	3.740
TeAb-K	22,620 (27,550–15,540)	0.1563 (0.1716–0.1429)	3536	2.57 (4.60–1.02)	1.138 (1.290–0.992)	2.924
LC-H_N_ (2G11 epitope)						
2G11	11.11 (13.75–8.46)	0.6317 (0.7363–0.5383)	7.018	11.41 (15.28–8.23)	0.5027 (5.514–4.559)	5.736
BeAb	21.13 (25.37–16.23)	0.3937 (.4378–.3525)	8.320	12.78 (21.52–6.62)	2.346 (2.927–1.842)	29.98
TeAb-K	25.79 (29.75–22.21)	0.2616 (0.2739–0.2508)	6.747	2.59 (5.78–0.57)	2.128 (2.779–1.615)	5.512
H_CC_ (RAZ1 epitope)						
RAZ1	1.33 (1.92–0.813)	10.02 (14.61–6.222)	13.33	1.73 (1.96–1.53)	15.89 (16.84–15.03)	27.49
TeAb-K	25.53 (45.03–12.23)	2.115 (2.331–1.919)	53.99	15.18 (25.62–8.10)	1.186 (1.284–1.092)	18.00

^a^ 95% confidence interval; ^b^ the entire heavy chain was used for these measurements; ^c^ *k_off_* was calculated from *k_on_* and K_D_, therefore no CI is provided.

## Supplementary Materials

The following are available online at http://www.mdpi.com/2072-6651/10/2/84/s1, Figure S1: Binding affinity measurement of the BeAb and TeAb by flow fluorimetry, Table S1: Primers used for TeAb construction and sequencing confirmation.

## References

[1] Hatheway C.H., Snyder J.D., Seals J.E., Edell T.A., Lewis G.E. (1984). Antitoxin levels in botulism patients treated with trivalent equine botulism antitoxin to toxin types A, B, and E. J. Infect. Dis..

[2] Hibbs R.G., Weber J.T., Corwin A., Allos B.M., El Rehim M.S.A., El Sharkawy S., Sarn J.E., McKee K.T. (1996). Experience with the use of an investigational F (ab′) 2 heptavalent botulism immune globulin of equine origin during an outbreak of type E botulism in Egypt. Clin. Infect. Dis..

[3] Arnon S.S., Schechter R., Maslanka S.E., Jewell N.P., Hatheway C.L. (2006). Human botulism immune globulin for the treatment of infant botulism. N. Engl. J. Med..

[4] Frankovich T.L., Arnon S.S. (1991). Clinical trial of botulism immune globulin for infant botulism. West J. Med..

[5] Cangene Corp., BAT® [Botulism Antitoxin Heptavalent (A, B, C, D, E, F, G)—(Equine)] Sterile Solution for Injection. https://www.fda.gov/downloads/.../UCM345147.pdf.

[6] Fagan R.P., Neil K.P., Sasich R., Luquez C., Asaad H., Maslanka S., Khalil W. (2011). Initial recovery and rebound of type f intestinal colonization botulism after administration of investigational heptavalent botulinum antitoxin. Clin. Infect. Dis. Off. Publ. Infect. Dis. Soc. Am..

[7] Nowakowski A., Wang C., Powers D.B., Amersdorfer P., Smith T.J., Montgomery V.A., Sheridan R., Blake R., Smith L.A., Marks J.D. (2002). Potent neutralization of botulinum neurotoxin by recombinant oligoclonal antibody. Proc. Natl. Acad. Sci. USA.

[8] Fan Y., Barash J.R., Lou J., Conrad F., Marks J.D., Arnon S.S. (2016). Immunological Characterization and Neutralizing Ability of Monoclonal Antibodies Directed Against Botulinum Neurotoxin Type H. J. Infect. Dis..

[9] Fan Y., Garcia-Rodriguez C., Lou J., Wen W., Conrad F., Zhai W., Smith T.J., Smith L.A., Marks J.D. (2017). A three monoclonal antibody combination potently neutralizes multiple botulinum neurotoxin serotype F subtypes. PLoS ONE.

[10] Nayak S., Griffiss J., McKenzie R., Fuchs E., Jurao R., An A., Ahene A., Tomic M., Hendrix C., Zenilman J. (2014). Safety and Pharmacokinetics of XOMA 3AB, a Novel Mixture of Three Monoclonal Antibodies against Botulinum Toxin A. Antimicrob. Agents Chemother..

[11] Wu C., Ying H., Grinnell C., Bryant S., Miller R., Clabbers A., Bose S., McCarthy D., Zhu R.R., Santora L. (2007). Simultaneous targeting of multiple disease mediators by a dual-variable-domain immunoglobulin. Nat. Biotechnol..

[12] Lou J., Geren I., Garcia-Rodriguez C., Forsyth C.M., Wen W., Knopp K., Brown J., Smith T., Smith L.A., Marks J.D. (2010). Affinity maturation of human botulinum neurotoxin antibodies by light chain shuffling via yeast mating. Protein Eng. Des. Sel..

[13] Razai A., Garcia-Rodriguez C., Lou J., Geren I., Forsyth C.M., Robles Y., Tsai R., Smith T.J., Amith L.A., Siegel R.W. (2005). Molecular evolution of antibody affinity for sensitive detection of botulinum neurotoxin type A. J. Mol. Biol..

[14] Meng Q., Li M., Silberg M.A., Conrad F., Bettencourt J., To R., Huang C., Ma J., Meyer K., Shimizu R. (2012). Domain-based assays of individual antibody concentrations in an oligoclonal combination targeting a single protein. Anal. Biochem..

[15] DiGiammarino E.L., Harlan J.E., Walter K.A., Ladror U.S., Edalji R.P., Hutchins C.W., Lake M.R., Greischar A.J., Liu J., Ghayur T. (2011). Ligand Association Rates to the Inner-Variable-Domain of a Dual-Variable-Domain Immunoglobulin are Significantly Impacted by Linker Design. MAbs.

[16] Strotmeier J., Mahrhold S., Krez N., Janzen C., Lou J., Marks J.D., Binz T., Rummel A. (2014). Identification of the synaptic vesicle glycoprotein 2 receptor binding site in botulinum neurotoxin A. FEBS Lett..

[17] Tu C., Terraube V., Tam A.S.P., Stochaj W., Fennell B.J., Lin L., Stahl M., LaVallie E.R., Somers W., Finlay W.J. (2016). A Combination of Structural and Empirical Analyses Delineates the Key Contacts Mediating Stability and Affinity Increases in an Optimized Biotherapeutic Single-chain Fv (scFv). J. Biol. Chem..

[18] Solomon H.M., Lilly T. Bacteriological Analytical Manual (BAM): Clostridium botulinum. https://www.fda.gov/Food/FoodScienceResearch/LaboratoryMethods/ucm070879.htm.

[19] Al-Saleem F.H., Ancharski D.M., Ravichandran E., Joshi S.G., Singh A.K., Gong Y., Simpson L.L. (2008). The role of systemic handling in the pathophysiologic actions of botulinum toxin. J. Pharmacol. Exp. Ther..

[20] Webb R.P., Smith T.J., Wright P., Brown J., Smith L.A. (2009). Production of catalytically inactive BoNT/A1 holoprotein and comparison with BoNT/A1 subunit vaccines against toxin subtypes A1, A2, and A3. Vaccine.

[21] Lacy D.B., Tepp W., Cohen A.C., DasGupta B.R., Stevens R.C. (1998). Crystal structure of botulinum neurotoxin type A and implications for toxicity. Nat. Struct. Biol..

[22] O’Hear C.E., Foote J. (2005). Antibody buffering of a ligand in vivo. Proc. Natl. Acad. Sci. USA.

[23] Mannik M., Haakenstad A.O., Arend W.P., Brent L., Holborow J. (1974). The fate and detection of circulating immune complexes. Progress in Immunology, II.

[24] Montero-Julian F.A., Klein B., Gautherot E., Brailly H. (1995). Pharmacokinetic study of anti-interleukin-6 (IL-6) therapy with monoclonal antibodies: Enhancement of IL-6 clearance by cocktails of anti-IL-6 antibodies. Blood.

[25] Garcia-Rodriguez C., Levy R., Arndt J.W., Forsyth C.M., Razai A., Lou J., Geren I., Stevens R.C., Marks J.D. (2007). Molecular evolution of antibody cross-reactivity for two subtypes of type A botulinum neurotoxin. Nat. Biotechnol..

[26] Garcia-Rodriguez C., Geren I., Lou J., Conrad F., Forsyth C., Wen W., Chakraborti S., Zao H., Manzanarez G., Smith T. (2011). Neutralizing human monoclonal antibodies binding multiple serotypes of botulinum neurotoxin. Protein Eng. Des. Sel..

[27] Blake R.C., Pavlov A.R., Blake D.A. (1999). Automated kinetic exclusion assays to quantify protein binding interactions in homogeneous solution. Anal. Biochem..

[28] Kaech S., Banker G. (2006). Culturing hippocampal neurons. Nat. Protoc..

[29] Fan Y., Geren I.N., Dong J., Lou J., Wen W., Conrad F., Smith T.J., Smith L.A., Ho M., Pires-Alves M. (2015). Monoclonal Antibodies Targeting the Alpha-Exosite of Botulinum Neurotoxin Serotype/A Inhibit Catalytic Activity. PLoS ONE.

[30] Ohi M., Li Y., Cheng Y., Walz T. (2004). Negative Staining and Image Classification—Powerful Tools in Modern Electron Microscopy. Biol. Proced. Online.

[31] Cheng L.W., Onisko B., Johnson E.A., Reader J.R., Griffey S.M., Larson A.E., Tepp W.H., Stanker L.H., Brandon D.L., Carter J.M. (2008). Effects of purification on the bioavailability of botulinum neurotoxin type A. Toxicology.

[32] Correia I., Sung J., Burton R., Jakob C.G., Carragher B., Ghayur T., Radziejewski C. (2013). The Structure of Dual-Variable-Domain Immunoglobulin Molecules Alone and Bound to Antigen. MAbs.

[33] Adekar S.P., Takahashi T., Jones R.M., Al-Saleem F.H., Ancharski D.M., Root M.J., Kapadnis B.P., Simpson L.L., Dessain S.K. (2008). Neutralization of botulinum neurotoxin by a human monoclonal antibody specific for the catalytic light chain. PLoS ONE.

[34] Dimasi N., Gao C., Fleming R., Woods R.M., Yao X.T., Shirinian L., Kiener P.A., Wu H. (2009). The design and characterization of oligospecific antibodies for simultaneous targeting of multiple disease mediators. J. Mol. Biol..

[35] Kurtz J.E., Dufour P. (2010). Adecatumumab: An anti-EpCAM monoclonal antibody, from the bench to the bedside. Expert Opin. Biol. Ther..

[36] Torisu-Itakura H., Schoellhammer H.F., Sim M.S., Irie R.F., Hausmann S., Raum T., Baeuerle P.A., Morton D.L. (2011). Redirected Lysis of Human Melanoma Cells by a MCSP/CD3-bispecific BiTE Antibody That Engages Patient-derived T Cells. J. Immunother..

[37] Vugmeyster Y., Zhang Y.E., Zhong X., Wright J., Leung S.S. (2014). Pharmacokinetics of anti-IL17A and anti-IL22 peptide–antibody bispecific genetic fusions in mice. Int. Immunopharmacol..

[38] Alt M., Müller R., Kontermann R.E. (1999). Novel tetravalent and bispecific IgG-like antibody molecules combining single-chain diabodies with the immunoglobulin γ1 Fc or CH3 region. FEBS Lett..

[39] Castoldi R., Jucknischke U., Pradel L.P., Arnold E., Klein C., Scheiblich S., Niederfellner G., Sustmann C. (2012). Molecular characterization of novel trispecific ErbB-cMet-IGF1R antibodies and their antigen-binding properties. Protein Eng. Des. Sel..

[40] Schoonjans R., Willems A., Schoonooghe S., Fiers W., Grooten J., Mertens N. (2000). Fab chains as an efficient heterodimerization scaffold for the production of recombinant bispecific and trispecific antibody derivatives. J. Immunol..

[41] Wang X.-B., Zhao B.-F., Zhao Q., Piao J.-H., Liu J., Lin Q., Huang H.-L. (2004). A new recombinant single chain trispecific antibody recruits T lymphocytes to kill CEA (carcinoma embryonic antigen) positive tumor cells in vitro efficiently. J. Biochem..

[42] Wong W.M., Vakis S.A., Ayre K.R., Ellwood C.N., Howell W.M., Tutt A.L., Cawley M.I., Smith J.L. (2000). Rheumatoid arthritis T cells produce Th1 cytokines in response to stimulation with a novel trispecific antibody directed against CD2, CD3, and CD28. Scand. J. Rheumatol..

[43] Somasundaram C., Sundarapandiyan K., Keler T., Deo Y.M., Graziano R.F. (1999). Development of a trispecific antibody conjugate that directs two distinct tumor-associated antigens to CD64 on myeloid effector cells. Hum. Antib..

[44] Mertens N., Schoonjans R., Willems A., Schoonooghe S., Leoen J., Grooten J. (2001). New Recombinant Bi-and Trispecific Antibody Derivatives. Novel Front. Prod. Compd. Biomed. Use.

[45] Xu L., Pegu A., Rao E., Doria-Rose N., Beninga J., McKee K., Lord D.M., Wei R.R., Deng G., Louder M. (2017). Trispecific broadly neutralizing HIV antibodies mediate potent SHIV protection in macaques. Science.

