# Supplementary material for: A Single Tri-Epitopic Antibody Virtually Recapitulates the Potency of a Combination of Three Monoclonal Antibodies in Neutralization of Botulinum Neurotoxin Serotype A
Source: Toxins (Basel). 2018 Feb 15;10(2):84. doi: 10.3390/toxins10020084 (PMC5848185; doi:10.3390/toxins10020084)
Supplement: Supplementary file 1 [file toxins-10-00084-s001.pdf]

Supplementary Materials: A Single Tri-Epitopic Antibody Virtually Recapitulates the Potency of a Combination of Three Monoclonal Antibodies in Neutralization of Botulinum Neurotoxin Serotype A

Jianlong Lou, Weihua Wen, Fraser Conrad, Qi Meng, Jianbo Dong, Zhengda Sun, Consuelo Garcia-Rodriguez, Shauna Farr-Jones, Luisa W. Cheng, Thomas D. Henderson, Jennifer L. Brown, Theresa J. Smith, Leonard A. Smith, Anthony Cormier and James D. Marks

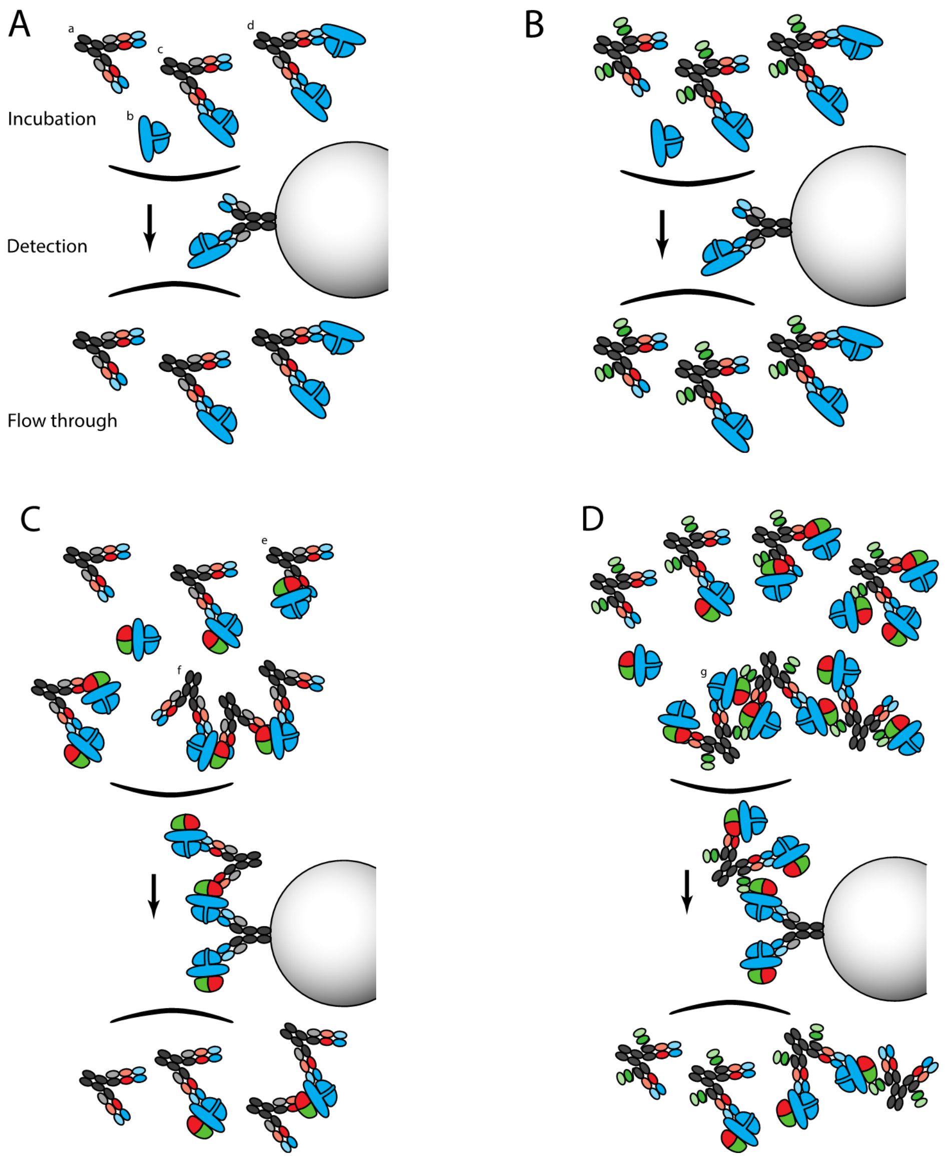

Figure S1. Binding affinity measurement of the BeAb and TeAb by flow fluorimetry. (**A**) During incubation of the BeAb with antibody-specific domains, 4 binding states exist: free antibody[a], free domain[b], and complexes where one or both of the binding sites of the antibody are occupied[c,d]. States with free epitopes (i.e. unbound domains) can bind to KinExA capture beads while others flow through; (**B**) Similarly, during incubation of the TeAb with antibody-specific domains 4 states exist and only unbound domain can bind to detection beads; (**C**) When the BeAb is incubated with holotoxin, the presence of multiple binding epitopes on the target molecule allows the formation of many more states, including avid binding at both sites[e] and crosslinking[f]. Any state with free target

epitopes may be captured by the beads; (**D**) When the TeAb is incubated with holotoxin, the presence of an additional binding site further supports the formation of large complexes[g].

Table 1: Primers used for TeAb construction and sequencing confirmation.

Name of Primer	Full Sequence (5′-)	Used for
ForTeAbscfvseq	CCTGCGAAGTCACCCATCAGG	RAZ1 scFv domain in TeAb sequencing
primer4scfvconfirm	GACAAACCACAACTAGAATGC	RAZ1 scFv domain in TeAb sequencing
TeAbK scFv seq3′primer	CGTTAACGGATCTGAATTCAACAC	RAZ1 scFv domain in TeAb sequencing
TeAbK scFv seq5'primer	GAAGTCACCCATCAGGGCCTGAGC	RAZ1 scFv domain in TeAb sequencing
TeAb2Vk seq5'primer	GTCAGATCGCCTGGAGACGCC	2G11&CR2 Vk in BeAb or TeAb sequencing
TeAb2Hlinker seq 3'primer	GGCCAGGGGGAACACGGAGGGTCC	2G11&CR2 VH in BeAb or TeAb sequencing
TeAb2Klinker seq 3'primer	GTCGGAAGGGGGAAAGATGAACACG	2G11&CR2Vk in BeAb or TeAb sequencing
DraIIIinBsiWoutVklinker3'primer	ACTGCTCATCACTGGGTGAGAAGATGAAGACA GATGGTGCAGCCACGGTACGTTTGATTTCC	2G11&CR2 Vk domain link subclone
BsiWIoutLinker1DraIIIin3'primer	CAACATCACATCGTGCACCTTTGGAAGAACCT TCACCAGACCCTGGCTTACCGGATCCGGAAGT AGATCCCGTTCGTTTTATTTCCACCTTGGTCCCC TGGC	2G11&CR2 Vk domain link subclone
Linker1Vk5'primer	CGAGGATCTACTTCCGGATCCGGTAAGCCAGG GTCTGGTGAAGGTTCTTCCAAAGGTGATGTTGT GATGACTCAGTCTCCATCC	2G11&CR2 Vk domain link subclone
Linker1Vk3'primer	ACCTTTGGAAGAACCTTCACCAGACCCTGGCT TACCGGATCCGGAAGTAGATCCTCGTTTTATTT CCACCTTGGTCCCCTG	2G11&CR2 Vk domain link subclone
BglIIinVk5'primer	GACGCCATCACAGATCTCTCACCATGAGGGTC CCCGCTCAGCTCCTGGGGCTCCTGCTGCTCTGG CTCCCAGGTGCCCGATGTCAGGTCCAGCTGCA GCAGTCTGGGGGAGG	2G11&CR2 Vk domain link subclone
2G11Vk3'end primer	GGAAGATGAAGACAGATGGTGCAGCCACCGT ACGTTTGATTTCCACCTTGGTCCCTCC	For 2G11&CR2 Vk domain link subclone
MfeIinMluIoutVH5'primer	ACCCGTCGGCAATTGCTCGACATGGGTTGGAG CCTCATCTTGCTCTTCCTTGTCGCTGTTGCTACC CGAGTCTTGTCCCAGGTCCAGCTGCAGCAGTC TGG	2G11&CR2 VH domain link subclone
LinkerMluIinVH3'primer	GGACAAGACACGCGTACCTTTGGAAGAACCTT CACCAGACCCTGGCTTACCGGATCCGGAAGTA GATCCCGTTCGTTTTATTTCCACCTTGGTCCCCT GG	2G11&CR2 VH domain link subclone
Linker2VH5'	CGAGGATCTACTTCCGGATCCGGTAAGCCAGG GTCTGGTGAAGGTTCTTCCAAAGGTCAGGTAC AGCTGCAGCAGTCAGGGGGAGGC	2G11&CR2 VH domain link subclone
2G11VH3'end primer	CGATGGGCCCTTGGTGCTAGCTGAGGAGACGG TGACCCGGGTTCC	2G11&CR2 VH domain link subclone
RAZVkNdeI5'primer	GCTCCCAGGTGCACATATGGACATCGTGATGA CCCAGTCTCC	RAZ1 scFv domain subclone to make TeAb
RAZlinkesplice3′primer	ACCTGGCTTACCGGAACCGGAAGTAGAACCTC CGGAACACTCTCCCCTGTTGAAGC	RAZ1 scFv domain subclone to make TeAb
RAZBsiWIlinkerVk5′primer	GAGATAAAACGTACGGTGGCTGCACCATCTGT CTTCATCTTCTCACGATGCGACATCGTGATGAC CCAG	RAZ1 Vk domain subclone to make TVD
Vk3′primer	GAAGCAAAGCTACTAGTCCCAGCTGG	RAZ1 Vk domain subclone to make TVD
RAZNheIlinkerVH5′ primer	TCCTCAGCTAGCACCAAGGGCCCATCAGTGAC GCGTGTCTTGTCCCAGGTGCAGCTGGTGCAGTC TGG	RAZ1 VH domain subclone to make TVD
VH3′ primer	CGACACCGTCACCGGTTCGGGGAAGTAGTCC	RAZ1 VH domain subclone to make TVD
LinkerHspliceRAZ5′primer	ACTTCCGGTTCCGGTAAGCCAGGTTCTGGTGA AGGTTCTTCTGGTTCCCAGGTGCAGCTGGTGCA GTCTGGG	RAZ1scFv domain preparation for TeAb-H from pYD2 vector
TeAbHBamHIin3′primer	TAGTTGGTAACCGTTAACGGATCCTCAACGTTT GATCTCCAGCTTGGTCCC	RAZ1scFv domain preparation for TeAb-H from pYD2 vector

TeAbH3 *Sma*I in 5′primer	GTGTACACCCTGCCCCCATCCCGGGATGAGCT GACCAAGAACCAGGTCAGCCTGACC	RAZ1scv domain subclone to make TeAb-H
RAZ1linkerH3splice 3′primer	ACCTGGCTTACCGGAACCGGAAGTAGAACCTC CGGATTTACCCGGAGACAGGGAGAGG	RAZ1scv domain subclone to make TeAb-H
LinkerKspliceRAZ5′primer	ACTTCCGGTTCCGGTAAGCCAGGTTCTGGTGA AGGTTCTTCTGGTTCCCAGGTGCAGCTGGTGCA GTCTGGG	RAZ1 scFv domain preparation for TeAb-K from pYD2 vector
TeAbKEcoRIin3′primer	CGTTAACGGATCTGAATTCAACGTTTGATCTCC AGCTTGGTCC	RAZ1 scFv domain preparation for TeAb-K from pYD2 vector
TeAbKBsiWIin5′primer	GGTACCAAGGTGGAAATCAAACGTACGGTGG CTGCACCATCTGTCTTCATCTTCC	RAZ1scv domain subclone to make TeAb-K
RAZlinkerKsplice 3′primer	ACCTGGCTTACCGGAACCGGAAGTAGAACCTC CGGAACACTCTCCCCTGTTGAAG	RAZ1scv domain subclone to make TeAb-K
